# Molecular antibiotic resistance mechanisms and co-transmission of the *mcr-9* and metallo-β-lactamase genes in carbapenem-resistant *Enterobacter cloacae* complex

**DOI:** 10.3389/fmicb.2022.1032833

**Published:** 2022-10-31

**Authors:** Shan Jiang, Xiaoyu Wang, Haidong Yu, Jisheng Zhang, Jianmin Wang, Jie Li, Xinhui Li, Kewang Hu, Xue Gong, Xuemei Gou, Yang Yang, Chunjiang Li, Xiaoli Zhang

**Affiliations:** ^1^Department of Microbiology, Yongchuan Hospital of Chongqing Medical University, Chongqing, China; ^2^Department of Pathogenic Biology, Jiamusi University School of Basic Medicine, Jiamusi, China; ^3^Department of Microbiology, The First Affiliated Hospital of Jiamusi University, Jiamusi, China; ^4^Department of Microbiology, Shenzhen University General Hospital, Shenzhen, China; ^5^Department of Microbiology, Affiliated Hangzhou Xixi Hospital, Zhejiang University School of Medicine, Hangzhou, China; ^6^Department of Life Science and Technology, Mudanjiang Normal University, Mudanjiang, China

**Keywords:** carbapenem-resistant *Enterobacter cloacae* complex, *mcr-9*, metallo-β-lactamase genes, whole-genome sequencing, IncHI2/2A, colistin

## Abstract

Carbapenem-resistant *Enterobacter cloacae* complex (CRECC) has increasingly emerged as a major cause of healthcare-associated infections, with colistin being one of the last-resort antibiotics of treatment. Mobile colistin resistance (*mcr*)*-9* is a member of a growing family of *mcr* genes and has been reported to be an inducible gene encoding an acquired phosphoethanolamine transferase. Here, we collected 24 ECC strains from Chongqing, China from 2018 to 2021. Subsequently, antibiotic resistance genes and the transmission dynamics of the strains were determined by PCR, whole-genome sequencing, and bioinformatic analysis. The *mcr-9* was identified in IncHI2/2A or IncHI2/2A + IncN plasmids from six CRECC strains and was co-located with *bla*_NDM-1_ or *bla*_IMP-4_ in 2/6 plasmids. The genetic environment of *mcr-9.1* was composed of IS*903B*-*mcr-9.1*-*wbuC*-IS*26* in the five *mcr-9.1*-harboring-plasmid, but IS*1B* was located downstream of *mcr-9.2* in the pECL414-1 sequence. We also found that the pNDM-068001 plasmid carrying *mcr-9.1* could be a hybrid plasmid, formed by a Tn*6360*-like *bla*_NDM-1_ region inserted into an *mcr-9.1*-positive IncHI2/2A plasmid. A conjugation assay showed that plasmids mediated the co-dissemination of *mcr-9* and metallo-β-lactamase (MBL) genes. In addition, we performed induction assays with sub-inhibitory concentrations of colistin and found an increase in the relative expression levels of the *mcr-9.2*, *qseC*, and *qseB* genes, as well as an increase in the minimum inhibitory concentration values of colistin in the CRECC414 strain. These findings provide a basis for studying the regulatory mechanisms of *mcr-9* expression and highlight the importance of effective monitoring to assess the prevalence of MBL and *mcr-9* co-existing plasmids.

## Introduction

The dramatic increase in the prevalence and clinical impact of infections caused by *Enterobacterales* producing carbapenemases, such as *Klebsiella pneumoniae* carbapenemase-2 (KPC-2) and New Delhi metallo-β-lactamase-1 (NDM-1), has triggered a global health crisis (Kumarasamy et al., 2010; Munoz-Price et al., 2013). Colistin belongs to the family of polymyxins, cationic polypeptides. Owing to its nephrotoxicity, the clinical use of colistin was discontinued in the 1980s; however, it was reintroduced in the 1990s because of the lack of effective antimicrobial agents for the treatment of multidrug-resistant Gram-negative pathogens (Falagas and Kasiakou, 2005). Previously reported mechanisms of colistin resistance are chromosomally mediated and involve the regulation of two regulatory systems (*pmrAB*, *phoPQ*, and its negative regulator *mgrB* in *K. pneumoniae*), resulting in the modification of lipid A by phosphoethanolamine or 4-amino-4-arabinose (Gunn, 2008; Cannatelli et al., 2013). However, a plasmid-mediated mobilized colistin resistance (*mcr*) gene *mcr-1* was first reported in China in late 2015 (Liu et al., 2016), which largely threatens the use of colistin in clinical settings, and nine *mcr* homologs (*mcr-2* to *-10*) have since been detected (Nang et al., 2019). Although the transmission of most *mcr* genes to carbapenem-resistant *Enterobacterales* (CRE) has been limited (Carroll et al., 2019; Kieffer et al., 2019; Nang et al., 2019), *mcr-9*, first identified in *Salmonella enterica* clinically isolated in the United States in 2019 (Carroll et al., 2019), has been identified in several CRE backgrounds (Chavda et al., 2019; Lalaoui et al., 2019; Liu et al., 2021). In addition, the first identified strain that carried *mcr-9* was sensitive to colistin, but *mcr-9* confers resistance to colistin after cloning and overexpression in the laboratory (Carroll et al., 2019). Further studies have revealed that *mcr-9* expression is inducible in the presence of colistin when *mcr-9* is located upstream of the *qseBC* two-component system (Kieffer et al., 2019). However, other studies have suggested that *mcr-9* and colistin resistance may not be related (Tyson et al., 2020). These results suggest that there is uncertainty as to when *mcr-9* confers elevated minimum inhibitory concentration (MIC) values for colistin, which may be related to the genetic background or differences in host and other unidentified regulatory genes (Chavda et al., 2019; Simoni et al., 2021).

In the present study, we investigated the antibiotic resistance genes (ARGs) and transmission dynamics of 24 ECC isolates collected from 2018 to 2021 using PCR, whole-genome sequencing (WGS), and bioinformatic analysis. We analyzed WGS data and compared them with publicly available data to determine the genome structure of strains carrying the *mcr-9* gene. We determined the phenotypic impact of carbapenem-resistant *Enterobacter cloacae* complex (CRECC) strains carrying *mcr-9* under the sub-inhibitory concentrations of colistin induction. Moreover, plasmid-mediated co-transmission of the *mcr-9* and MBL genes was determined using a conjugation assay.

## Materials and methods

### Bacterial isolates, susceptibility testing, and clinical data collection

*Enterobacter cloacae* complex (ECC) strains that were resistant to meropenem, imipenem, or ertapenem or produced carbapenemase isolated from 2018 to 2021 at Yongchuan Affiliated Hospital of Chongqing Medical University, China, were collected, in addition to one carbapenem-sensitive *Enterobacter cloacae* complex (CSECC) strain carrying *mcr-9*, identified in the screening, which was also included in this study. All 24 ECC strains were identified using a VITEK-2 Compact automatic microbiology analyzer and MALDI-TOF MS (Bruker, Billerica, MA, United States). Antimicrobial susceptibility was evaluated for all isolates following the Clinical and Laboratory Standards Institute (CLSI) guidelines (M100-S30; CLSI, 2020), and the results were interpreted according to these guidelines (Clinical and Laboratory Standards Institute (CLSI), 2020), except that colistin resistance was defined according to the European Committee on Antimicrobial Susceptibility Testing (EUCAST; version 10.0) clinical breakpoints (The European Committee on Antimicrobial Susceptibility Testing, 2020). Metadata including patients’ departments, dates of specimen collection, and specimen types were recorded.

### PCR and sequencing

Total DNA was extracted from each strain of the 24 ECC strains using the boiling method and used as a template in PCR experiments (Gong et al., 2018). Resistance genes were detected by PCR, including colistin-resistant genes (*mcr-1* and *mcr-9*), carbapenemase genes (*bla*_NDM_, *bla*_KPC_, *bla*_OXA-48_, *bla*_VIM-1_, *bla*_VIM-2_, *bla*_IMP-4_, and *bla*_IMP-8_), AmpC β-lactamase enzyme genes (*bla*_ACC_ and *bla*_DHA_), ESBL genes (*bla*_TEM_, *bla*_SHV_, *bla*_CTX-M-1_, and *bla*_CTX-M-9_), and quinolone resistance genes (*qnrA*, *qnrB*, *qnrS*, *qepA*, and *aac(6′)Ib-cr*). All primers have been used in previous studies (Park et al., 2006; Ma et al., 2009; Gong et al., 2018). Positive amplification products were subjected to Sanger sequencing, and the obtained sequences were subsequently submitted to Basic Local Alignment Search Tool (BLAST).

### Whole-genome sequencing and bioinformatic analysis

WGS was performed using an Illumina HiSeq PE150 (Illumina, San Diego, CA, United States) on the 24 ECC isolates, with six CRECC isolates carrying *mcr-9* proceeding to further long-read sequencing (Oxford Nanopore, Oxford, United Kingdom). Long-read sequencing assembly was performed using the unicycler v0.4.8 (Wick et al., 2017), and the correction was performed using the pilon v1.22 (Walker et al., 2014). *In silico* multi-locus sequence typing (MLST) was performed using PubMLST^1^ (Jolley et al., 2018). The plasmid replicon type and plasmid MLST (pMLST) were identified using PlasmidFinder 2.1,^2^ and pMLST 2.0^3^ (Carattoli et al., 2014). ResFinder 4.1^4^ was employed for the identification of ARGs (Bortolaia et al., 2020). Complete plasmid sequence alignments were performed using BLAST and visualized using the BRIG tool. Sequence alignments among *mcr-9*-carrying or *qseC-qseB*-carrying plasmids or chromosomes were performed using BLAST and visualized using Easyfig v 2.2.5.

A total of 2,337 complete genomes of ECC strains were retrieved from the NCBI database (accessed on 31 December 2021) and were subjected to comparative alignments of *mcr-9* positive plasmid genomes isolated from patients using BLAST and visualized using Easyfig v 2.2.5.

### Core genome MLST and cg-SNP

All strains contained 3,048 core genomes according to the reference genome (GenBank accession no. CP010377). The results of core genome MLST (cg-MLST) were visualized using PHYLOViZ, based on the goeBURST algorithm (Ribeiro-Goncalves et al., 2016). The results of core genome single nucleotide polymorphism (cg-SNP) were visualized using ggtree after building the evolutionary tree with FastMe (Lefort et al., 2015).

### Conjugation experiment

We selected six isolates of CRECC carrying the *mcr-9* and carbapenemase genes and used them as donors in a conjugation experiment, with rifampicin-resistant *Escherichia coli* EC600 as the recipient. Conjugation experiments were performed using the membrane bonding method according to previously described method (Gong et al., 2018). Briefly, donor (CRECC strain) and recipient cultures (*E. coli* EC600) were mixed at a 1:3 ratio in Luria-Bertani (LB) broth. The mixtures were placed on a membrane and subsequently incubated at 35°C for 24 h. Mueller–Hinton agar plates were supplemented with meropenem (1 mg/l) and rifampicin (600 mg/l) to select transconjugants. The VITEK-2 Compact system and 16S rRNA sequence were performed to confirm the transconjugants and PCR was determined to the presence of resistance genes.

### Colistin induction assays

Colistin induction assays were performed as previously described with some modifications (Kieffer et al., 2019; Liu et al., 2021). Briefly, the CRECC414 strain was inoculated in 4 ml LB broth with shaking at 37°C for 2 h and then supplemented with colistin (1 and 2 mg/l) or without at a final bacterial suspension of 1.0 McFarland turbidity standard. After shaking at 37°C for 6 h, bacterial suspensions were used for mRNA extraction.

### mRNA extraction and RT-qPCR

Total RNA was extracted using the PureLink^TIM^ RNA Mini Kit (Thermo Fisher Scientific), as specified by the manufacturer. Each RNA sample was reverse-transcribed using the PrimeScript™ RT reagent kit (TaKaRa, Japan) according to the manufacturer’s instructions. The relative expression level of *mcr-9* was determined by quantitative real-time PCR (RT-qPCR) using SYBR Green detection reagents on the CFX96 RealTime PCR system. The primer sequences used are listed in Supplementary Table 1. Relative expression levels were calculated using the 2^-ΔΔCT^ method, with *rpoB* as a reference gene for comparison with those of samples that were not induced. Three independent replicates were performed with and without induction.

### Nucleotide sequence accession number

WGS data were submitted to NCBI with accession numbers in Supplementary Table 2.

## Results

### Isolate characteristics

**Figure 1:**
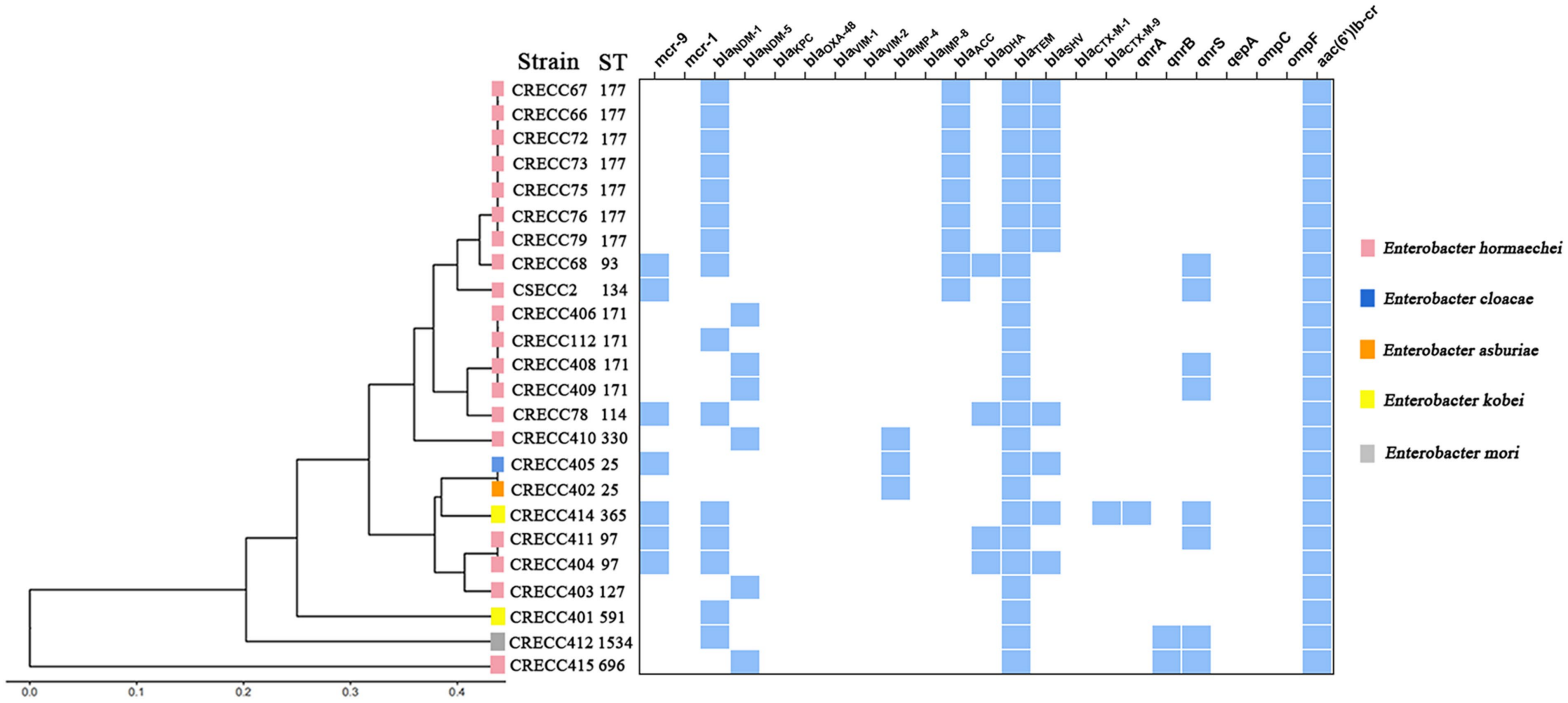
Clustering analysis and resistance determinants of 24 ECC strains from our institution. The resulting structure reflected the similarity between the sequences, and resistance determinants present (in blue) in each strain are shown on the right.

**Figure 2:**
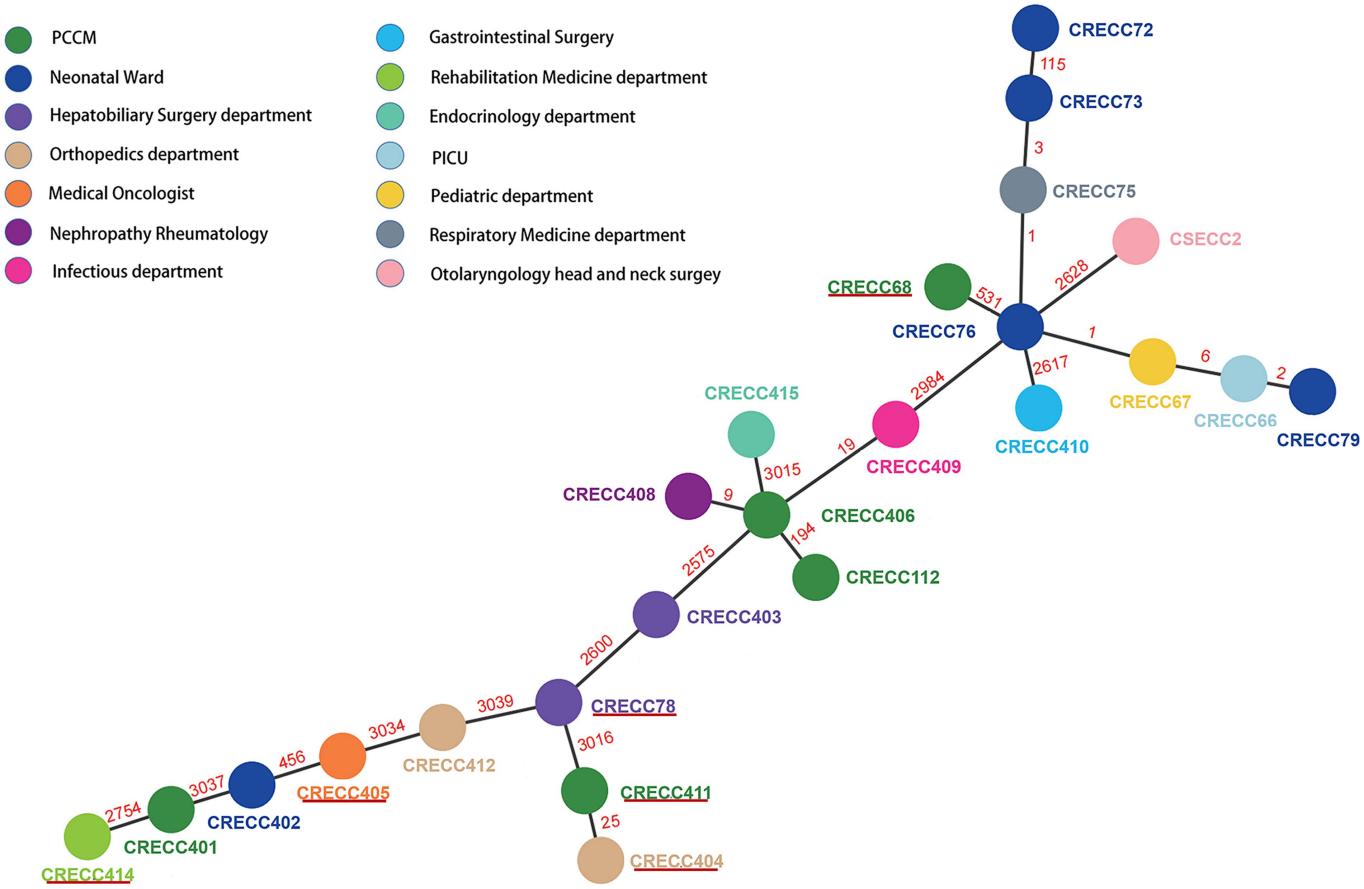
Minimum spanning tree for multilocus sequence typing of the core genome based on 3,084 targets. Allelic distances between isolates are represented numerically. The resulting structure reflected the similarity between strains isolated by different departments. Solid red lines represent CRECC strains carrying *mcr-9*.

We collected 24 non-repetitive clinical ECC isolates, 23 of which were CRECC strains and 1 CSECC strain (Supplementary Table 2). In total, 24 ECC isolates were identified to five species: *Enterobacter hormaechei* (*n* = 19), *Enterobacter kobei* (*n* = 2), *Enterobacter cloacae* (*n* = 1), *Enterobacter asburiae* (*n* = 1), and *Enterobacter mori* (*n* = 1; Figure 1). Regarding the source of the strains, sputum accounted for the highest proportion (54.2%; 13/24), followed by urine (12.5%; 3/24), blood (12.5%; 3/24), and bronchoalveolar lavage fluid (12.5%; 3/24). Notably, at least nine isolates (37.5%; 9/24) were recovered from sterile site specimens of patients (Supplementary Table 2). All 23 CRECC strains carried the MBL genes (*bla*_NDM-1_*, bla*_NDM-5,_ or *bla*_IMP-4_, except for the CRECC410 strain carrying both *bla*_NDM-5_ and *bla*_IMP-4_), the ESBL gene (*bla*_TEM-1_), and the quinolone resistance gene (*aac(6’)Ib-cr*; Figure 1). Among them, six CRECC strains (CRECC68, CRECC78, CRECC404, CRECC405, CRECC411, and CRECC414) carried *mcr-9* on IncHI2-ST1 plasmids and carried *qseC-qseB-*like two-component regulatory system on the chromosomes. The six CRECC strains belonged to three species: *E. hormaechei* (66.6%; 4/6), *E. kobei* (16.7%, 1/6), and *E. cloacae* (16.7%, 1/6; Figure 1). According to the MLST analysis, 24 ECC strains showed that the main ST types were ST177 (CRECC72, CRECC73, CRECC75, CRECC76, CRECC67, CRECC66, and CRECC79) and ST171 (CRECC406, CRECC408, CRECC409, and CRECC112; Figure 1). Based on the cg-MLST results, there were 1–115 allelic differences between the ST177 strains, as well as an average nucleotide identity (ANI) of more than 99.9% (Figure 2 and Supplementary Figure 1), which further illustrates the similarity between strains. In addition, the ANI among ST171 strains was more than 99.9%, but the ANI results between CRECC112 and other ST171 strains ranged from 99.82 to 99.88% (Supplementary Figure 1). Phylogenetic analysis of cg-SNP also showed similarities between CRECC406, CRECC408, and CRECC409 (Supplementary Figure 2). Therefore, this may represent the clonal dissemination of ST177 strains between the PICU, neonatal ward, respiratory medicine department, and pediatric department of our institution from October 21, 2018, to June 6, 2019 (Figures 1, 2; Supplementary Table 2).

### Transferability of *mcr-9*-carrying plasmids

The *mcr-9* genes of CRECC68 and CRECC405 strains co-occurred on the same plasmids as *bla*_NDM-1_ and *bla*_IMP-4,_ respectively. However, *mcr-9* and *bla*_NDM-1_ of other CRECC strains (CRECC78, CRECC404, CRECC411, and CRECC414) were located on plasmids of different sizes (Supplementary Table 3). Meropenem was used for screening and six *E. coli* EC600 transconjugants were identified using VITEK-2, 16S rRNA, and PCR. The six transconjugants were found to contain the MBL genes (*bla*_NDM-1_ or *bla*_IMP-4_) and the colistin-resistant genes (*mcr-9*; Supplementary Table 3), and were resistant to meropenem and rifampicin. This suggests that using meropenem may promote the co-transmission of *mcr-9* and MBL genes, which could lead to a more serious public health crisis.

### Genetic environment of *mcr-9*-carrying isolates

**Figure 3:**
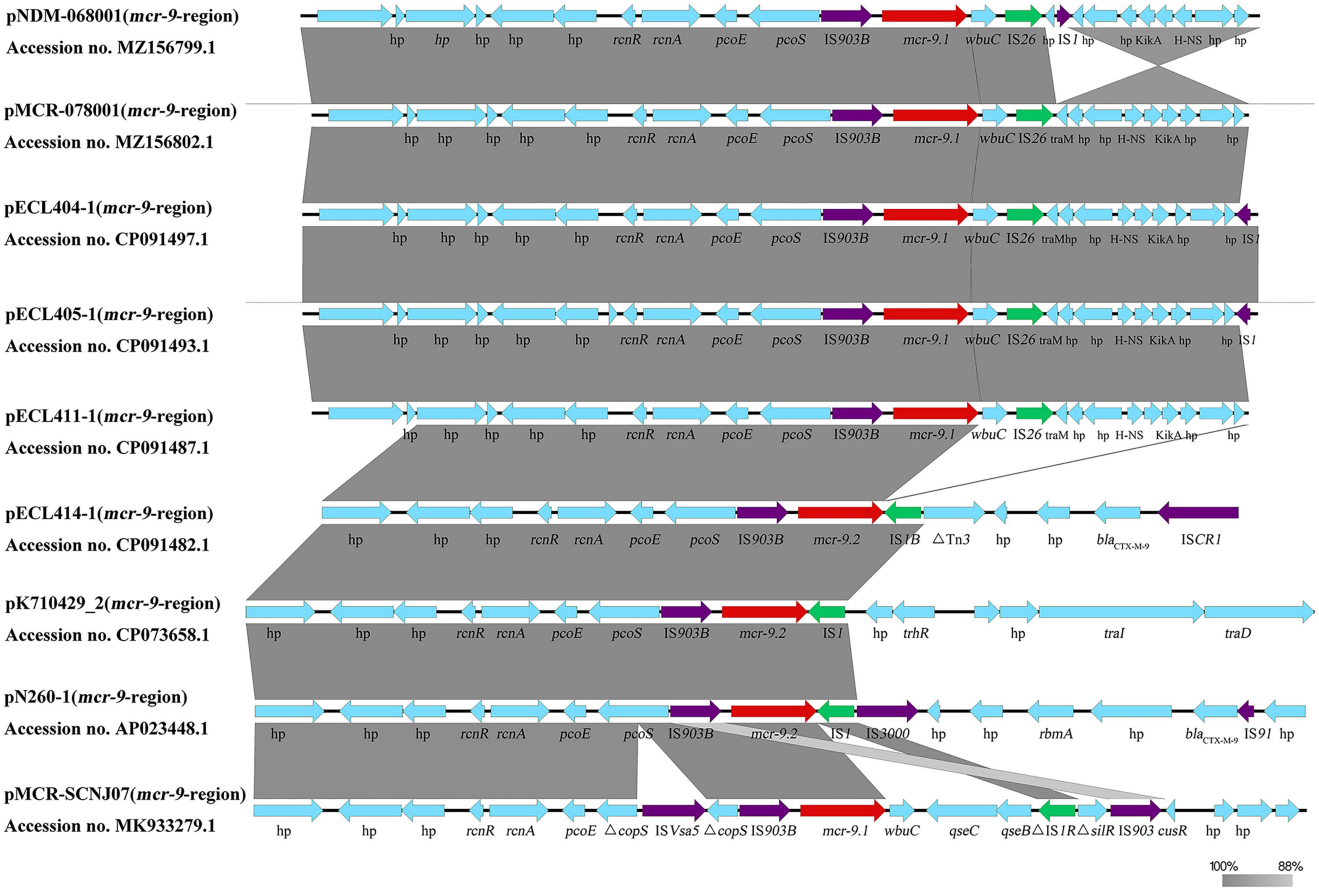
Different genetic structure surrounding the *mcr-9* gene. Comparison of *mcr-9* regions detected in pNDM-068001, pMCR-078001, pECL404-1, pECL405-1, pECL411-1, pECL414-1, pN260-1, pKPN7104292-2, and pMCR-SCNJ07. Gray shading denotes regions of shared homology. Arrows indicate the direction of gene transcription.

We identified 26 *mcr-9*-containing complete plasmid genomes isolated from patients in the NCBI database (Supplementary Figure 3). We found that most (24/26) of the *mcr-9* genetic environments showed 100% homology to the backbone composed of *rcnR-rcnA-pcoE-pcoS-*IS*903B-mcr-9*. Among them, more than half (19/24) were found to have *wbuC-*IS*26* downstream of *mcr-9*, and two (2/24) plasmid sequences were found to have *qseC-qseB-*IS*1* downstream of *mcr-9*. Another two (2/24) plasmid sequences were found where *pcoS* was interrupted by ISVsa5. It is suggested that *mcr-9* may be transferred as a gene cassette (*rcnR-rcnA-pcoE-pcoS-*IS*903B-mcr-9-wbuC-*IS*26*) among the plasmids. In the present study, *mcr-9* was located in an almost conserved region in the five *mcr-9*-harboring plasmids (pNDM-068001, pMCR-078001, pECL404-1, pECL405-1, and pECL411-1). *wbuC* was downstream of *mcr-9.1* and *mcr-9.1-wbuC* was surrounded upstream by an IS*903B* and downstream by an IS*26*; however, comparative alignments of the genetic environments found that IS*1B* was located downstream of *mcr-9.2* in the pECL414-1 sequence (Figure 3).

### Analysis of *mcr-9*-harboring plasmids from our institution

**Figure 4:**
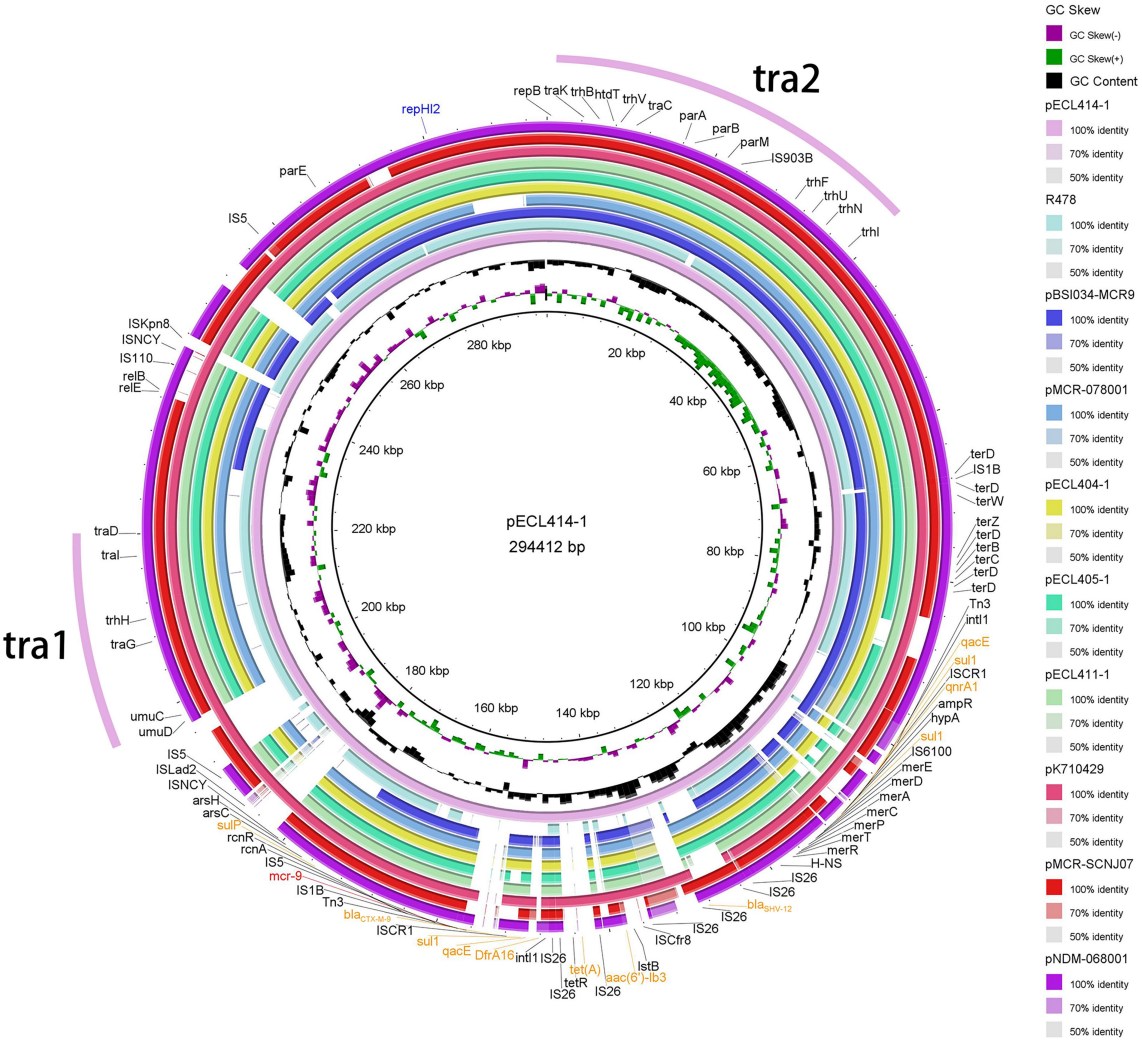
Plasmid structure of *mcr-9.* Sequence alignment of *mcr-9*-harboring plasmids pNDM-068001, pMCR-078001, pECL404-1, pECL405-1, pECL411-1, pECL414-1, pR478, pK710429, pMCR-SCNJ07, and pBSI034-MCR9. The complete plasmid pECL414-1 sequence was used as the reference, and the white and colored regions of the circles indicate absence and presence, respectively. Antibiotic resistance genes are shown in orange (*mcr-9* is shown in red), and the tra1 and tra2 regions for conjugative transfer are indicated by pink curves on the outer circle.

In our setting, *mcr-9* was found to be located on one IncHI2/2A + IncN plasmid and five different IncHI2/IncHI2A plasmids, named pNDM-068001, pMCR-078001, pECL404-1, pECL405-1, pECL411-1, and pECL414-1, with sizes of 444,489, 342,942, 319,000, 362,923, 316,592 and 294,412 bp, respectively. pECL414-1 shared 99.97% identity and 84% coverage with plasmid pMCR-SCNJ07 carried by *E. hormaechei* in Neijiang, Sichuan, which was identified in 2019, and pECL414-1 shared 100% identity and 97% coverage with plasmid pK710429 carried by *K. pneumoniae* in Anhui, Sichuan, which was identified in 2021. Comparison of the six *mcr-9*-carrying plasmids revealed that with a coverage range of 81–100%, they shared at least 99% identity, whereas the genome sequence of pNDM-068001 to pECL414-1 showed a lower coverage of 71% and nucleotide identity of 99.52%. This showed that they shared a similar backbone that mostly included regions essential for plasmid replication, maintenance, and conjugative transference (Figure 4). Except for *mcr-9*, multiple ARGs located on *mcr-9*-harboring plasmids were mainly responsible for the multidrug resistance phenotype of the isolates (Supplementary Tables 2, 4). These included *bla*_NDM-1_, *bla*_IMP-4_, *bla*_DHA-1_, *bla*_SHV-12_, *bla*_CTX-M-9_, and *bla*_TEM-1_ for β-lactam resistance; *strAB*, *aph(3′)-Ia*, *aac(6′)-llc*, and *aac(6′)-lb* for aminoglycoside resistance; *dfrA19*, *dfrA16*, and *dfrA14* for trimethoprim resistance; *sul1* for sulfonamide resistance; *tet(D)* and *tet(A)* for tetracycline resistance; *qnrS1*, *qnrA1*, and *qnrB4* for quinolone resistance; and *ere(A)* and *mph(A)* for macrolide resistance (Supplementary Table 4). This prompts that *mcr-9* was frequently present on mobile genetic elements with other ARGs, implying that the use of these types of antibiotics has the potential to co-select for the continued presence of *mcr-9*.

### Colistin induction assays and colistin susceptibility testing

**Figure 5:**
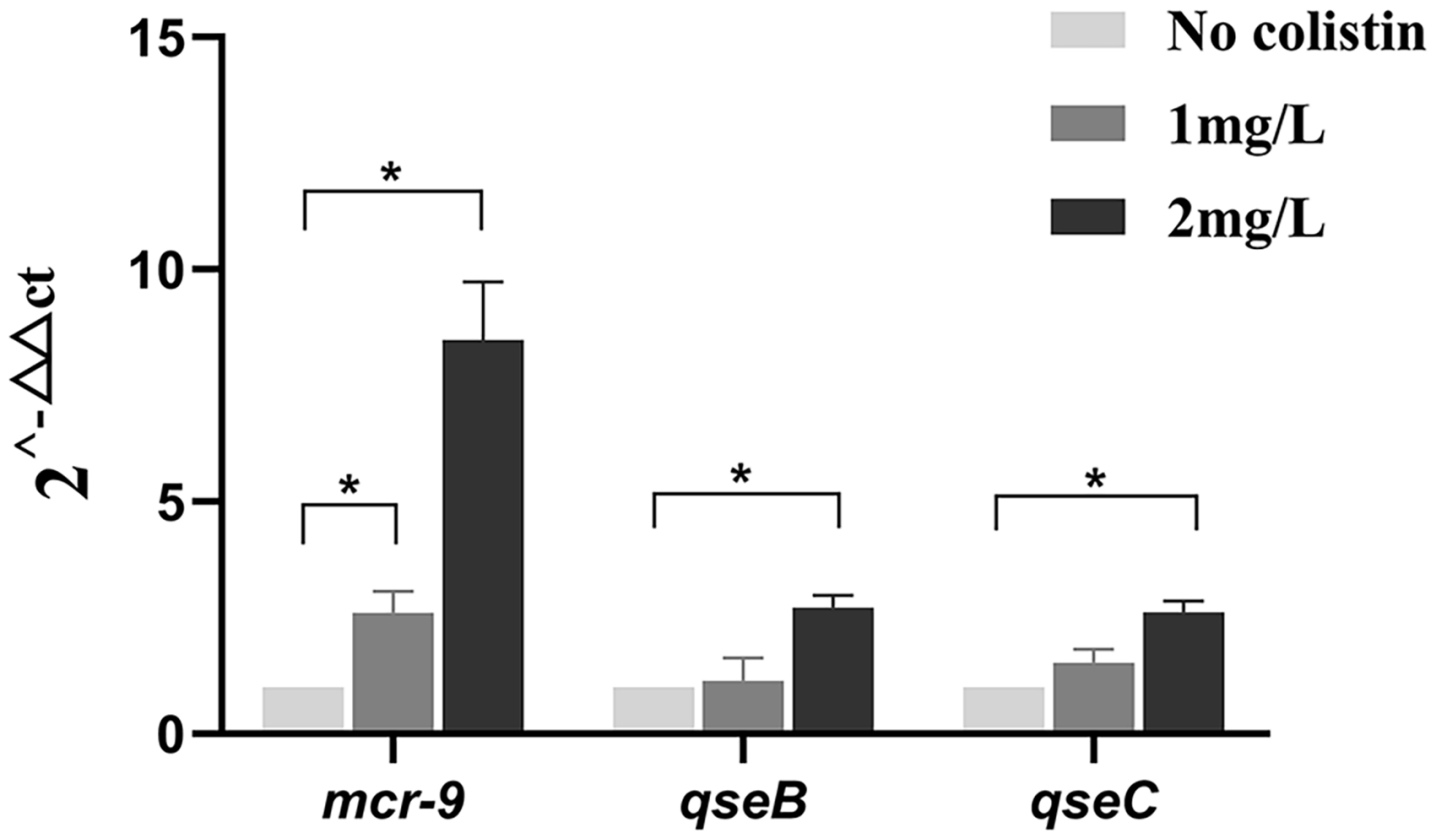
Expression of genes in clinical isolate CRECC414 with and without colistin exposure. The genes are the plasmid-located *mcr-9* and the chromosome-located *qseB* and *qseC*. Fold changes in mRNA levels were determined by RT-qPCR. The data have been normalized to values for reference *rpoB* gene. Three independent replicates were performed and the data shown represent the means. The asterisks indicate statistical significance at different levels by Student’s *t*-tests: ^*^p ≤ 0.05.

The strains were exposed to sub-inhibitory concentrations of colistin, and the CRECC414 strain was used as the template for mRNA extraction. Notably, a 2.6-fold increase in the plasmid-located *mcr-9* mRNA was detected once the culture was induced with 1 mg/l colistin; however, there was no statistically significant difference in the relative expression levels of the *qseB* and *qseC* genes located on chromosomes (Figure 5). After induction with 2 mg/l colistin, the relative expression levels of *mcr-9*, *qseC*, and *qseB* mRNA increased by 8.5-, 2.6-, and 2.7-fold, respectively (Figure 5). In parallel, the MIC value of colistin for the CRECC414 strain was 4 mg/l, and the MIC values were 64 and 128 mg/l after induction with 1 or 2 mg/l colistin, respectively.

## Discussion

The IncHI2-ST1 plasmid is the main replicon type carrying *mcr-9*, which is a member of a growing family of mobile colistin resistance genes. The *mcr-9* is predominantly present in MBL-producing strains at our institution; therefore, novel beta-lactam/beta-lactamase inhibitor combinations have no activity against them. The use of colistin in CRE infections remains a priority. However, the present study found an increase in the MIC value of strains *mcr-9*-carrying for colistin under antibiotic selection pressure, which may raise concerns regarding the use of colistin.

We performed phylogenetic analysis of plasmids carrying *mcr-9* from published studies and our institution. The results showed that 26 plasmids had the IncHI2 replicon alone, one had IncHI2 + IncR, one had IncHI2 + IncM1, and one had IncHI2 + IncN (Supplementary Figure 4). This suggests that different replicon-type plasmids (IncHI2, IncHI2 + IncR, and IncHI2 + IncN) may represent important vehicles for mediating the dissemination of *mcr-9* in China. In addition, IncHI2 and IncHI2 + IncN were the main replicon-type in our setting, which may be frequently involved in the spread of multiple ARGs (Chowdhury et al., 2019; Borjesson et al., 2020; Supplementary Tables 3, 4). The IncHI2-IncN hybrid plasmid, pNDM-068001 (accession no. MZ156799.1), clustered with several IncHI2 plasmids (accession no. CP080514.1, MN937241.1, CP059887.1, and AP024500.1), implying that they may have a common ancestor and a subsequent genetic recombination event, leading to the formation of hybrid plasmids. The results of the conjugation assay showed that *mcr-9-*encoding plasmids can be horizontally transmitted between different species of *Enterobacterales*. Phylogenetic analysis also showed that *mcr-9-*encoding plasmids have been identified in several types of strains, with ECC isolates being the main isolates that disseminate *mcr-9* (Li et al., 2020; Supplementary Figure 4). Notably, *mcr-9*-harbouring plasmids recovered from human isolates were interspersed with plasmids from animals and food (Supplementary Figure 4), and it is possible that the use of colistin in food animals largely contributes to the prevalence of *mcr* genes in humans and the environment (Sun et al., 2018). Interestingly, we found that *mcr-9* genetic environments were major composed of IS*903B*-*mcr-9*-*wbuC*-IS*26* in our setting (Figure 3), and this genetic environment has been reported previously (Wu et al., 2022). In addition, few reports have described *mcr-9* integration into the chromosome of *E. hormaechei* (Wu et al., 2022), *Citrobacter* (Ribeiro et al., 2021), and *Salmonella* isolates (Tyson et al., 2020; Pan et al., 2021) with a genetic context similar to that observed in *mcr-9*-harboring plasmid sequences, suggesting that *mcr-9* may be transferred between plasmids and chromosomes in the form of a gene cassette.

According to MLST typing, six CRECC isolates carrying *mcr-9* were ST97 (33.3%, 2/6), ST93, ST114, ST25, and ST365 (Figure 1), which are most likely horizontal transmission rather than clonal transmission in our hospital. Interestingly, molecular epidemiology revealed that ST93 is the predominant sequence type of the CRECC strain, followed by ST171 and ST145 in China (Chen et al., 2021); and ST114 is a common global CRECC strain clone (Peirano et al., 2018). The results of the current study showed that the CRECC68 and CRECC78 strains carrying *mcr-9* belonged to ST93 or ST114, indicating that *mcr-9* may have co-evolved with the host, which carries a high risk of transmission.

Studies have been conducted on the phenotypic effects of strains under antibiotic selection pressure. Kieffer et al. (2019) found that the expression of *mcr-9* is inducible by subinhibitory concentrations of colistin and is related to the presence of *qseC* and *qseB* genes downstream of *mcr-9.* However, the MIC values of a few isolates in the presence of the *qseB/qseC*-like two-component system downstream of *mcr-9* remained unchanged after colistin exposure (Kim et al., 2021; Macesic et al., 2021; Simoni et al., 2021). It was also found that the absence of *qseC* and *qseB* genes downstream of *mcr-9* also does not change the MIC values of the strains after colistin exposure (Chavda et al., 2019; Bitar et al., 2020; Wu et al., 2022). The different results could be related to differences in the genetic background, host, and other undiscovered regulatory genes (Chavda et al., 2019; Simoni et al., 2021). In the present study, six CRECC strains carrying *mcr-9* were isolated from six patients from different departments, all of whom had different diseases, but none of them had been treated with colistin (Supplementary Table 5). First, we found that the colistin resistance rate of six CRECC strains carrying *mcr-9* was 50% (Supplementary Table 2). Nevertheless, it has been reported that few isolates carrying *mcr-9* display colistin resistance at baseline (Carroll et al., 2019; Kieffer et al., 2019; Tyson et al., 2020; Macesic et al., 2021). Alignment revealed no possible mutations in chromosomal resistance genes in our strains. Second, the relative expression levels of *mcr-9.2* and MIC values of the CRECC414 strain increased after colistin exposure, which is consistent with the results of *K. pneumoniae* (Liu et al., 2021). Although *qseC* and *qseB* are not located downstream of *mcr-9.2* on the plasmid, our experiments showed an increase in the relative expression levels of *qseC* and *qseB* after colistin induction. Therefore, we hypothesized that once *mcr-9* is integrated into the chromosome, it may be regulated by the *qseB/qseC*-like two-component of the *lysM-mdaB-qseC-qseB-parC-parF-sufl* genetic background (Supplementary Figure 5), ultimately leading to a possible increase in the relative expression levels of *mcr-9* and the MIC value of colistin in the CRECC strain under antibiotic selection pressure. In addition, comparative alignments of the genetic environments revealed that IS*1B* was located downstream of *mcr-9.2* in both the CRECC414 and *K. pneumoniae* strains (Figure 3). Similarly, IS*1* was found downstream of *mcr-9* in both pN260-1 and pMCR-SCNJ07 sequences, and these strains displayed colistin resistance at baseline (Yuan et al., 2019; Umeda et al., 2021; Figure 3). We hypothesized that the induced expression of *mcr-9* is related to the presence of downstream IS*1*. Therefore, further validation of these possible regulatory mechanisms will be the focus of future research.

The strength of the present study was the extensive use of Illumina and Nanopore read sequencing to construct high-quality hybrid plasmid assemblies. Notably, two *mcr-9*-harboring plasmids also carried *bla*_IMP-4_ (pECL405-1) or *bla*_NDM-1_ (pNDM-068001), respectively (Supplementary Table 3). We found that *bla*_NDM-1_ and *mcr-9* were located on the IncHI2/2A + IncN plasmid, and the *bla*_NDM-1_ cassette was similar to the recently reported cassette of Tn*6360* (carrying *bla*_NDM-1_ and located on the IncN1 plasmid; Zhao et al., 2017), suggesting that pNDM-068001 could be a hybrid plasmid, formed by a Tn6360-like *bla*_NDM-1_ region inserted into an *mcr-9*-positive IncHI2/HI2A plasmid. In addition, *bla*_IMP-4_ and *mcr-9* were located on the IncHI2/2A plasmid, and *bla*_IMP-4_ was characterized by the following structure: IS*6-dfrA19-IntI1-bla_IMP-4_-ltrA-SMR-sul1-GNAT-*IS*6* (IS*6100*), which may mediate the transfer of *bla*_IMP-4._ This indicated that horizontal gene transfer events played a significant role in plasmid evolution. Notably, the results of our study showed a co-transmission characteristic of the *mcr-9* and MBL genes, and colistin for CRE infections remains an important priority. We also found that *mcr-9* was inducible in the CRECC strains; therefore, this may pose some challenges for the use of colistin in the clinical environment. The current study has a minor limitation; the boiling method extracts a small amount of DNA with many impurities, and DNA breakage may occur, leading to errors. However, PCR and sequencing of the extracted nucleic acids for the primary screening of ARGs in strains still have some value.

In conclusion, the spread of *mcr-9* is primarily driven by the IncHI2/2A plasmid. Our results alert physicians to the circulation of plasmid-mediated co-transmission of *mcr-9* and MBL genes already in the hospital setting, which could lead to a serious public health crisis; therefore, effective monitoring is urgently needed to assess the prevalence of MBL and *mcr-9* co-existing plasmids and to find an effective measure to control their spread. We found that *mcr-9* showed increased expression when induced with colistin in the CRECC strain, which may be related to IS downstream of *mcr-9* or *qseB/qseC*-like two-component systems on chromosomes. Further clarification of their regulatory mechanisms for *mcr-9* gene expression will be the focus of our future research.

## Data availability statement

The data presented in the study are deposited at GenBank, accession number(s) can be found in the article/Supplementary material. Further inquiries can be directed to the corresponding author.

## Author contributions

XZ, CL, SJ, and XW conceived and designed the study. SJ and XW wrote the manuscript and participated in the whole experiment process. XL and KH helped with the experimental process. SJ, XW, HY, JZ, JW, and JL analyzed and interpreted the data. JW, JL, XGong, XGou, YY, SJ, and XW collected the isolates and the clinical data. All authors contributed to the article and approved the submitted version.

## Funding

This work was supported by General projects of Chongqing Natural Science Foundation (cstc2020jcyj-msxm0067), Yongchuan Natural Science Foundation (2021yc-jckx20053), Talent introduction project of Yongchuan Hospital of Chongqing Medical University (YJYJ202004 and YJYJ202005), and Program for Youth Innovation in Future Medicine, Chongqing Medical University (W0113).

## Conflict of interest

The authors declare that the research was conducted in the absence of any commercial or financial relationships that could be construed as a potential conflict of interest.

## Publisher’s note

All claims expressed in this article are solely those of the authors and do not necessarily represent those of their affiliated organizations, or those of the publisher, the editors and the reviewers. Any product that may be evaluated in this article, or claim that may be made by its manufacturer, is not guaranteed or endorsed by the publisher.

## Footnotes

^1^ https://pubmlst.org/

^2^ https://cge.food.dtu.dk/services/PlasmidFinder/

^3^ https://cge.food.dtu.dk/services/pMLST/

^4^ https://cge.food.dtu.dk/services/ResFinder/
