# Supplementary material for: Molecular antibiotic resistance mechanisms and co-transmission of the mcr-9 and metallo-β-lactamase genes in carbapenem-resistant Enterobacter cloacae complex
Source: Front Microbiol. 2022 Oct 31;13:1032833. doi: 10.3389/fmicb.2022.1032833 (PMC9659896; doi:10.3389/fmicb.2022.1032833)
Supplement: Supplementary file 1 [file Data_Sheet_1.docx]

**Supplementary Table 1**

Oligonucleotide sequences used in this work

| Primer | Sequence | Application |
| --- | --- | --- |
| *mcr-9* | Forward, 5’-CGGTACCGCTACCGCAATAT-3’  Reverse,5’-ATAACAGCGAGACACCGGTT-3’ | RT-qPCR |
| *qseC* | Forward,5’-AGCAGGACAATCAGCAACAGCAG-3’  Reverse,5’-CAGGAGTGGGAGTACCGACAGG-3’ | RT-qPCR |
| *qseB* | Forward,5’-ATCAAGCACCACGGCATCATACG-3’  Reverse,5’-CATTGGCGACGGCATCAAAACG-3’ | RT-qPCR |
| *rpoB* | Forward,5’-AAGGCGAATCCAGCTTGTTCAGC-3’  Reverse,5’-TGACGTTGCATGTTCGCACCCATCA-3’ | RT-qPCR |

RT-qPCR: quantitative real-time PCR.

**Supplementary Table 2**

Characterization of ECC strains identified in this study

| Isolates | Specimen | Date of isolation | MIC^a^ | | | | | | | | | | | | | | |  |
| --- | --- | --- | --- | --- | --- | --- | --- | --- | --- | --- | --- | --- | --- | --- | --- | --- | --- | --- |
|  |  |  | COL | MEM | IMP | TZP | CZO | CRO | CAZ | FEP | ATM | CIP | LVX | TOB | AMK | GEN | SMZ-  TMP | NCBI  accession |
| CRECC401 | Bronchoalveolar lavage fluid | 30/5/2021 | 2 | 32 | ≥16 | 64 | ≥64 | ≥64 | ≥64 | 32 | 8 | ≥4 | ≥8 | 4 | ≤2 | ≥16 | ≤20 | JAMYDI000000000 |
| CRECC402 | Sputum | 24/9/2020 | 2 | 4 | ≥16 | 8 | ≥64 | ≥64 | ≥64 | ≥64 | ≤1 | ≤0.25 | ≤0.25 | 8 | ≤2 | 2 | ≤20 | JAMYDH000000000 |
| CRECC403 | Blood | 6/6/2021 | 2 | 32 | ≥16 | ≥128 | ≥64 | ≥64 | ≥64 | ≥64 | ≥64 | ≥4 | 4 | ≥16 | ≤2 | ≤1 | ≥320 | JAMYDG000000000 |
| CRECC404 | Drainage fluid | 5/10/2020 | 1 | 16 | 8 | ≤4 | ≥64 | ≥64 | ≥64 | ≥64 | ≥64 | 0.5 | 1 | ≥16 | 32 | ≤1 | 80 | CP091496-  CP091503 |
| CRECC405 | Bronchoalveolar lavage fluid | 27/3/2021 | >128 | 8 | 8 | ≤4 | ≥64 | ≥64 | ≥64 | ≥64 | ≥64 | 0.5 | 1 | ≥16 | ≥64 | ≤1 | 80 | CP091492-  CP091495 |
| CRECC406 | Sputum | 23/10/2020 | 2 | 32 | 8 | ≥128 | ≥64 | ≥64 | ≥64 | ≥64 | ≤1 | ≥4 | ≥8 | 8 | ≤2 | ≤1 | ≥320 | JAMYDF000000000 |
| CRECC408 | Sputum | 14/7/2021 | 2 | 16 | 8 | 64 | ≥64 | ≥64 | ≥64 | ≥64 | ≤1 | ≥4 | ≥8 | 8 | ≤2 | ≥16 | ≥320 | JAMYDE000000000 |
| CRECC409 | Urine | 2/6/2021 | 2 | 16 | ≥16 | 64 | ≥64 | ≥64 | ≥64 | ≥64 | ≤1 | ≥4 | ≥8 | 8 | ≤2 | ≥16 | ≥320 | JAMYDD000000000 |
| CRECC410 | Bronchoalveolar lavage fluid | 1/12/2020 | 2 | 32 | ≥16 | ≥128 | ≥64 | ≥64 | ≥64 | ≥64 | ≥64 | ≤0.25 | ≤0.25 | ≤1 | ≤2 | ≤1 | ≤20 | JAMYDC000000000 |
| CRECC411 | Urine | 13/12/2020 | 1 | 64 | >8 | >16/8 | >16 | >32 | >16 | >16 | 8 | >2 | >4 | >8 | ≤16 | >8 | >2/38 | CP091486-  CP091491 |
| CRECC412 | Blood | 2/9/2020 | 2 | 16 | 8 | 8 | 64 | ≥64 | ≥64 | ≥64 | ≥64 | ≤0.25 | ≤0.25 | ≤2 | 8 | ≥16 | ≥320 | JAMYDB000000000 |
| CRECC414 | Sputum | 13/10/2021 | 4 | 16 | 8 | 64 | ≥64 | ≥64 | ≥64 | ≥64 | ≥64 | ≤0.25 | ≤0.25 | 4 | ≤2 | 4 | ≤20 | CP091481-  CP091485 |
| CRECC415 | Blood | 14/10/2021 | 2 | 16 | ≥16 | 64 | ≥64 | ≥64 | ≥64 | ≥64 | ≤1 | ≥4 | ≥8 | 8 | ≤2 | ≥16 | ≥320 | JAMYDA000000000 |
| CRECC66 | Sputum | 1/3/2019 | 2 | 16 | ≥16 | ≥128 | ≥64 | ≥64 | ≥64 | ≥64 | ≥64 | 0.5 | 1 | 8 | ≤2 | ≥16 | ≥320 | JAMYCZ000000000 |
| CRECC67 | Sputum | 15/5/2019 | 2 | 32 | 8 | ≥128 | ≥64 | ≥64 | ≥64 | ≥64 | ≥64 | 0.5 | 1 | ≤1 | ≤2 | ≤1 | ≤20 | JAMYCY000000000 |
| CRECC68 | Sputum | 11/5/2019 | 4 | 64 | 4 | ≥128 | ≥64 | ≥64 | ≥64 | ≥64 | ≥64 | ≥4 | ≥8 | 8 | ≤2 | ≥16 | ≥320 | CP088932,  MZ156799 |
| CRECC72 | Sputum | 16/2/2019 | 2 | 64 | ≥16 | ≥128 | ≥64 | ≥64 | ≥64 | ≥64 | ≥64 | ≥4 | ≥8 | 8 | ≤2 | ≥16 | ≥320 | CP077659，MZ156800-  MZ156801 |
| CRECC73 | Sputum | 5/2/2019 | 4 | 64 | ≥16 | ≥128 | ≥64 | ≥64 | ≥64 | ≥64 | ≥64 | ≥4 | 4 | 8 | ≤2 | ≥16 | ≥320 | JAMYCX000000000 |
| CRECC75 | Urine | 19/12/2018 | 4 | 16 | 8 | ≥128 | ≥64 | ≥64 | ≥64 | ≥64 | ≥64 | ≥4 | ≥8 | 16 | 8 | ≤1 | ≥320 | JAMYCW000000000 |
| CRECC76 | Sputum | 19/12/2018 | 4 | 32 | ≥16 | ≥128 | ≥64 | ≥64 | ≥64 | ≥64 | ≥64 | 0.5 | 1 | 8 | ≤2 | ≥16 | ≥320 | JAMYCV000000000 |
| CRECC78 | Bile | 23/9/2018 | 2 | 8 | 8 | >64 | >16 | >32 | >16 | >16 | >16 | >2 | >4 | >8 | 32 | >8 | >2/38 | CP077660,  MZ156802-  MZ156803 |
| CRECC79 | Sputum | 12/11/2018 | 2 | 32 | ≥16 | ≥128 | ≥64 | ≥64 | ≥64 | ≥64 | ≥64 | 0.5 | 1 | 8 | ≤2 | ≥16 | ≥320 | JAMYCU000000000 |
| CRECC112 | Sputum | 11/6/2020 | 1 | 4 | 4 | 64 | >32 | >32 | >32 | >32 | 16 | >2 | >4 | >8 | >32 | >8 | - | CP077661,  MZ156804 |
| CSECC  2 | Sputum | 12/9/2021 | 2 | ≤1 | ≤1 | ≤4 | ≤4 | ≤1 | ≤1 | ≤1 | ≤1 | ≤0.25 | ≤0.25 | ≤1 | ≤2 | ≤1 | ≤20 | JAMYDJ000000000 |

a, mg/L; COL, Colistin; MEM, Meropenem; IMP, Imipenem; TZP, Piperacillin-Tazobactam; CZO, Cefazolin; CRO, ceftriaxone; CAZ, Ceftazidime; FEP, Cefepime; ATM, Aztreonam; CIP, Ciprofloxacin; LVX, levofloxacin; TOB, Tobramycin; AMK, Amikacin; GEN, Gentamicin; SMZ-TMP, sulfamethoxazole-trimethoprim.

**Supplementary Table 3**

Characterization of *mcr-9*-positive CRECC and transconjugant strains identified in this study

| Isolates | Resistance genes^a^ | MIC^b^ | Inc type | | Isolates^e^ | Resistance genes^a^ | MIC |
| --- | --- | --- | --- | --- | --- | --- | --- |
|  |  | COL | *mcr-9*^c^ | Carbapenemase^d^ |  |  | COL |
| CRECC68 | *mcr-9.1*,  *bla*_NDM−1_ | 4 | IncHI2/2AIncN | - | J68 | *mcr-9.1*, *bla*_NDM−1_ | 2 |
| CRECC78 | *mcr-9.1*, *bla*_NDM−1_ | 2 | IncHI2/2A | IncX3 | J78 | *mcr-9.1*, *bla*_NDM−1_ | 2 |
| CRECC404 | *mcr-9.1*,  *bla*_NDM−1_ | 1 | IncHI2/2A | IncFII(Yp) | J404 | *mcr-9.1*, *bla*_NDM−1_ | 2 |
| CRECC405 | *mcr-9.1*,  *bla*_IMP-4_ | ＞128 | IncHI2/2A | - | J405 | *mcr-9.1*, *bla*_IMP-4_ | 2 |
| CRECC411 | *mcr-9.1*,  *bla*_NDM−1_ | 1 | IncHI2/2A | IncFII(Yp) | J411 | *mcr-9.1*, *bla*_NDM−1_ | 2 |
| CRECC414 | *mcr-9.2*,  *bla*_NDM−1_ | 4 | IncHI2/2A | IncX3 | J414 | *mcr-9.2*,  *bla*_NDM−1_ | 2 |

a, *mcr* gene and carbapenemase genes b, mg/L; c, *mcr-9*-harboring plasmid; d, carbapenemase-gene-harboring plasmid; e, *E. coli* transconjugant strains; “-”, co-occurrence of carbapenemase genes and *mcr-9* in a plasmid; COL, Colistin.

**Supplementary Table 4**

Distribution of resistance genes in 6 *mcr-9*-harboring plasmids.

| Plasmid name | | | pNDM-068001 | pMCR-078001 | pECL404-1 | pECL405-1 | pECL411-1 | pECL414-1 |
| --- | --- | --- | --- | --- | --- | --- | --- | --- |
| Host strains | | | CRECC68 | CRECC78 | CRECC404 | CRECC405 | CRECC411 | CRECC414 |
| Size (bp) | | | 444,489 | 342,942 | 319,000 | 362,923 | 316,592 | 294,412 |
| Resistance  genes | β-lactams | *bla*_NDM-1_ |  |  |  |  |  |  |
|  |  | *bla*_IMP-4_ |  |  |  |  |  |  |
|  |  | *bla*_DHA-1_ |  |  |  |  |  |  |
|  |  | *bla*_SHV-12_ |  |  |  |  |  |  |
|  |  | *bla*_CTX-M-9_ |  |  |  |  |  |  |
|  |  | *bla*_TEM-1_ |  |  |  |  |  |  |
|  | Aminoglycosides | *strAB* |  |  |  |  |  |  |
|  |  | *aph(3')-Ia* |  |  |  |  |  |  |
|  |  | *aac(6')-llc* |  |  |  |  |  |  |
|  |  | *aac(6')-lb* |  |  |  |  |  |  |
|  | Trimethoprims | *dfrA19* |  |  |  |  |  |  |
|  |  | *dfrA16* |  |  |  |  |  |  |
|  |  | *dfrA14* |  |  |  |  |  |  |
|  | Sulfonamides | *sul1* |  |  |  |  |  |  |
|  | Tetracyclines | *tet(D)* |  |  |  |  |  |  |
|  |  | *tet(A)* |  |  |  |  |  |  |
|  | Quinolones | *qnrS1* |  |  |  |  |  |  |
|  |  | *qnrA1* |  |  |  |  |  |  |
|  |  | *qnrB4* |  |  |  |  |  |  |
|  | Macrolides | *ere(A)* |  |  |  |  |  |  |
|  |  | *mph(A)* |  |  |  |  |  |  |
|  | Colistin | *mcr-9* |  |  |  |  |  |  |

^a^ Rectangles marked in red indicate the presence of the corresponding genes listed on the left.

**Supplementary Table 5**

Summary of clinical information for the *mcr-9*-carrying CRECC isolates

| Isolate | Department | Age | Gender | Diagnosis | Antibiotic usage |
| --- | --- | --- | --- | --- | --- |
| CRECC68 | PCCM | 65yr | Male | Chronic obstructivepulmonary disease, type 2 respiratoryfailure | Piperacillin/  tazobactam,  cefazolin |
| CRECC78 | Hepatobiliary Surgery department | 85yr | Male | Calculus of common bile duct | Ceftizoxime |
| CRECC404 | orthopedics department | 48yr | Male | Comminuted fracture of the right tibial plateau | Cefuroxim,amikacin,  piperacillin/  tazobactam |
| CRECC405 | Medical Oncologist | 52yr | Male | Lung cancer | Piperacillin |
| CRECC411 | PCCM | 82yr | Male | Bronchial asthma | Piperacillin/  tazobactam,  cefepime |
| CRECC414 | Rehabilitation Medicine  department | 72yr | Female | Traumatic brain injury | Ceftazidim,  levofloxacin |
