# Supplementary material for: Molecular antibiotic resistance mechanisms and co-transmission of the mcr-9 and metallo-β-lactamase genes in carbapenem-resistant Enterobacter cloacae complex
Source: Front Microbiol. 2022 Oct 31;13:1032833. doi: 10.3389/fmicb.2022.1032833 (PMC9659896; doi:10.3389/fmicb.2022.1032833)
Supplement: Supplementary file 2 [file Data_Sheet_1.PDF]

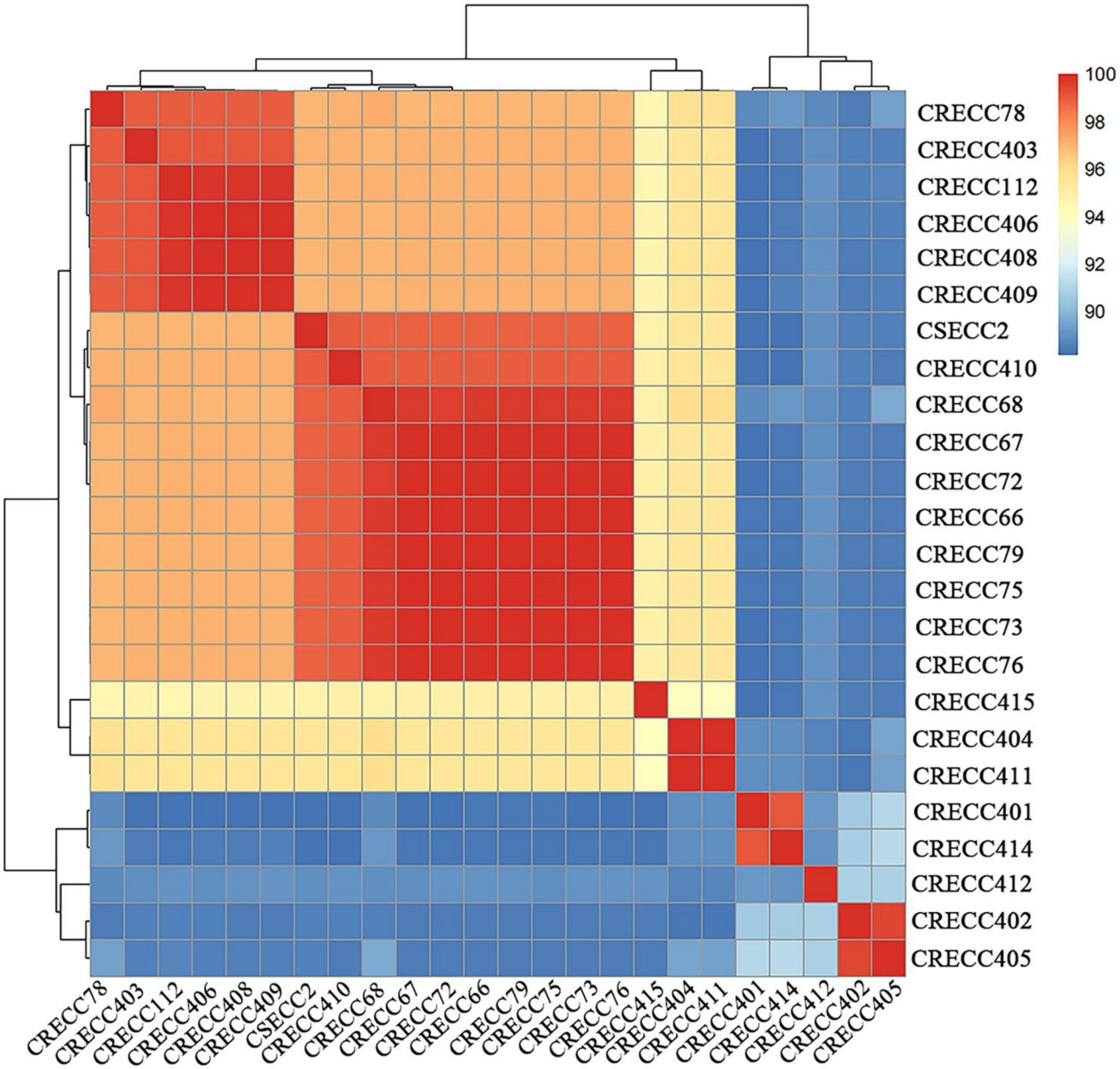

Supplementary Figure 1 Heat map based on average nucleotide identity (ANI) values between each pair of genome sequences from 24 ECC strains.

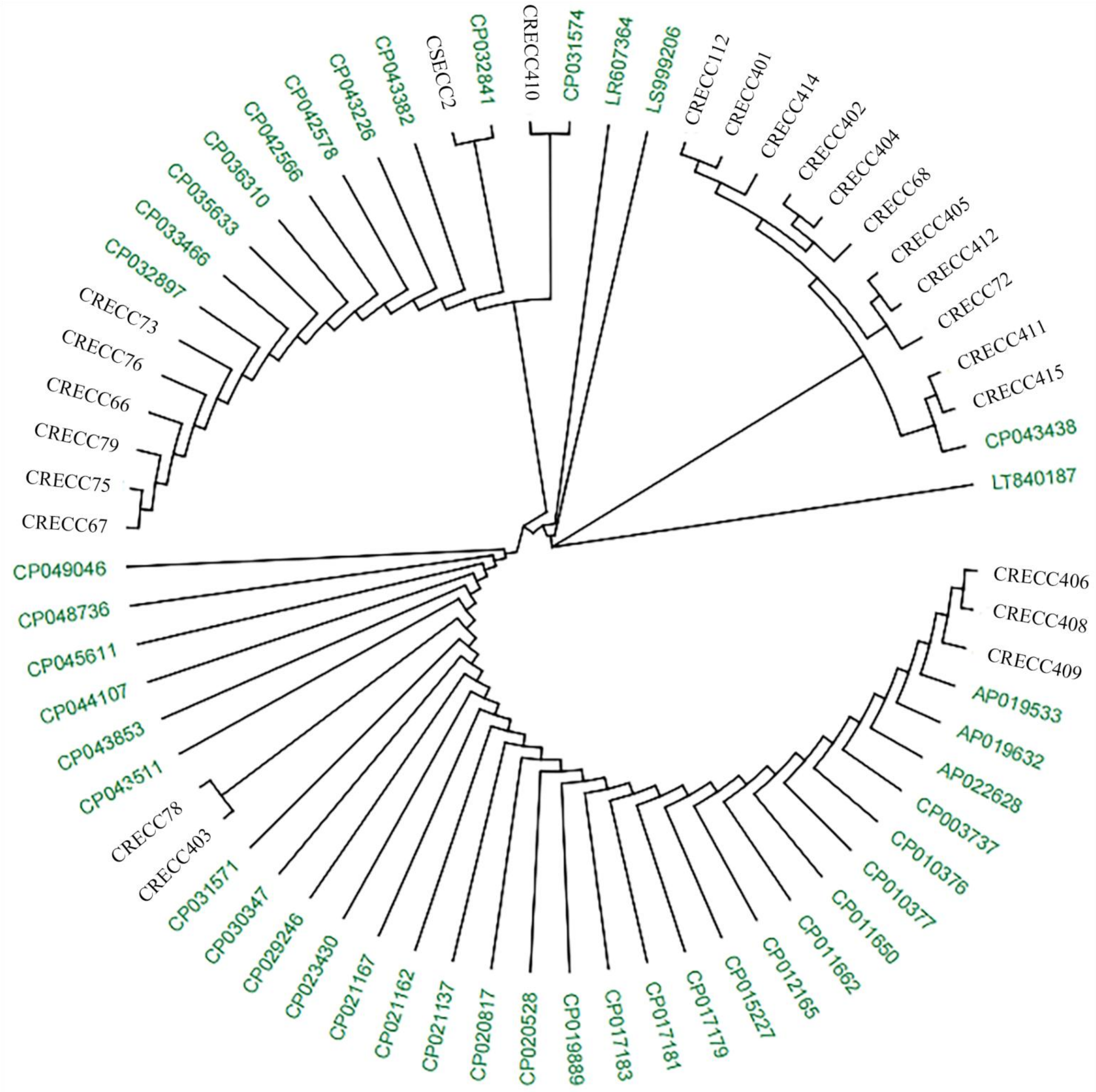

Supplementary Figure 2 Phylogenetic tree based on cg-SNP.

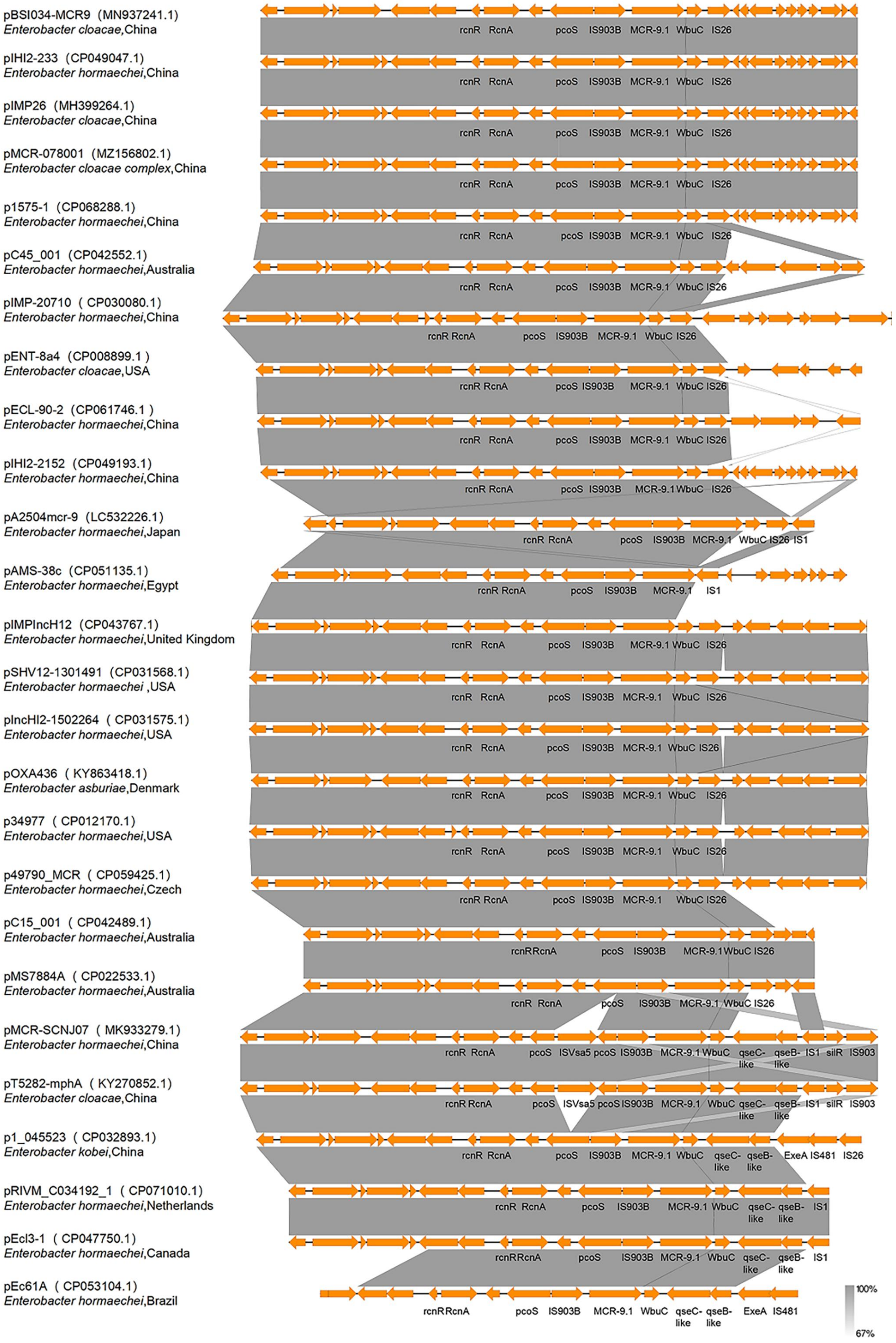

Supplementary Figure 3 Alignment of representative plasmids harbouring *mcr-9* gene. We performed BLASTn search of *mcr-9* in all (2337) complete genomes of ECC in NCBI database (Access on 31 December, 2021) and identified 26 *mcr-9*-containing complete plasmid genomes isolated from patients. Gray shading denotes regions of shared homology. Arrows indicate the direction of gene transcription.

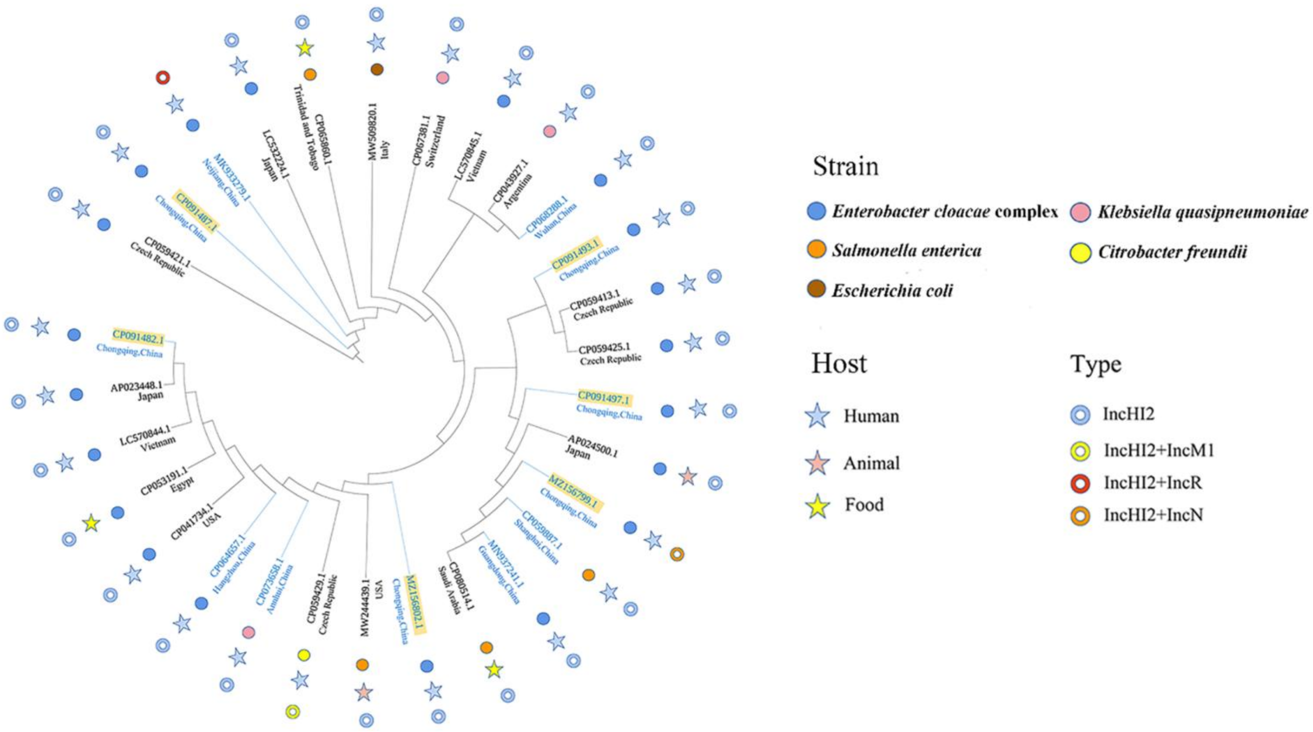

Supplementary Figure 4 Phylogenetic analysis of IncHI2 plasmids harbouring *mcr-9*. Plasmids harbouring *mcr-9* from published and our institution isolates were clustered on the basis of sequences similarity. Solid blue lines represent strains isolated from China and yellow background represent our strains.

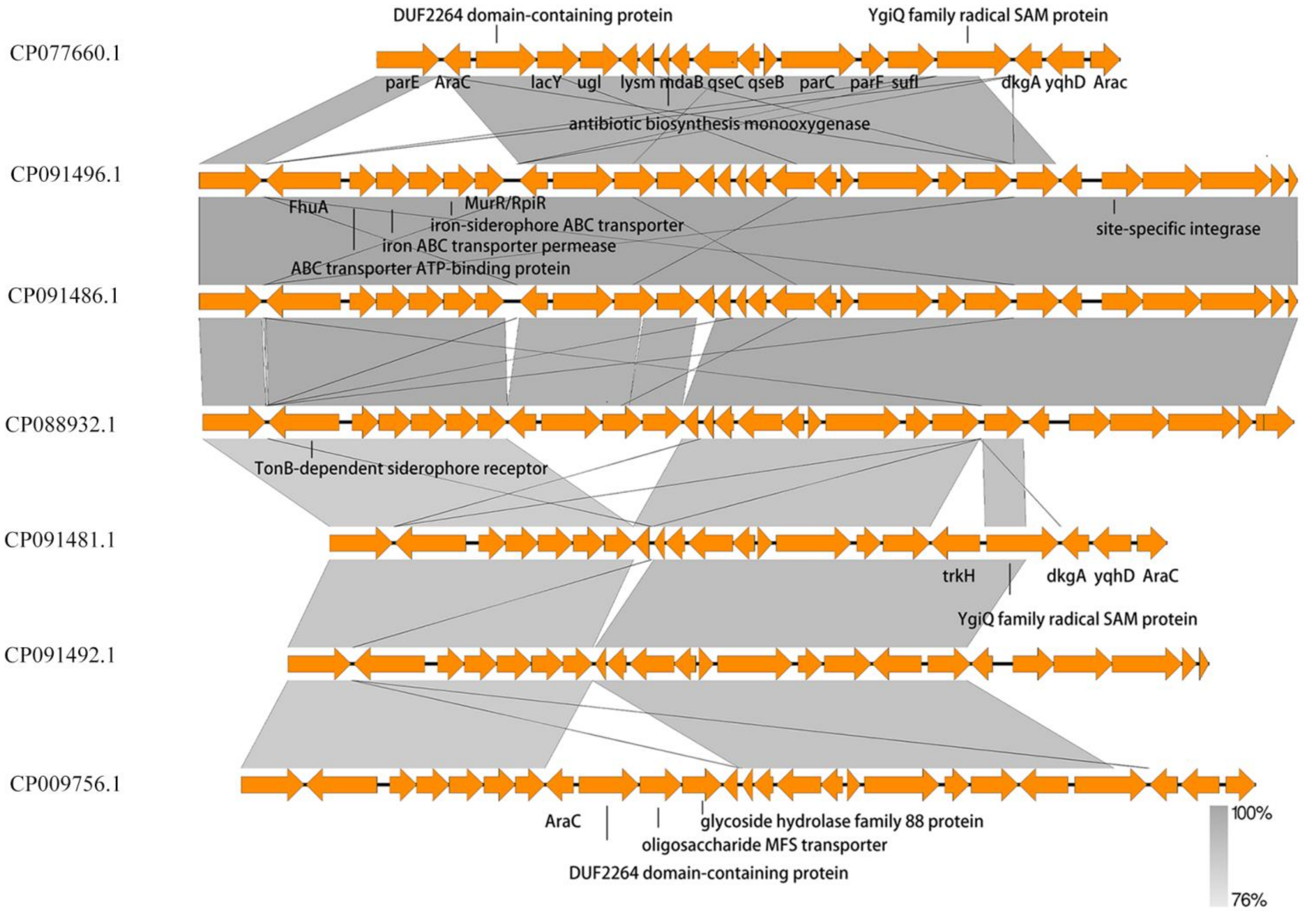

Supplementary Figure 5 Alignment of representative chromosome sequences harbouring *qseC* and *qseB* genes in our institution. The chromosome of GGT036 strain (accession no. CP009756.1) was used as a reference. Gray shading denotes regions of shared homology. Arrows indicate the direction of gene transcription.
